# Case Report: Hidden danger in breast eczematous lesions: cutaneous metastasis from silent lung adenocarcinoma misdiagnosed as Paget’s disease

**DOI:** 10.3389/fonc.2026.1911784

**Published:** 2026-08-19

**Authors:** Zhenni Gong, Yusheng Chen, Boyang Xue, Yali Zhang, Haozheng Cheng, Xuesong Jia, Su Liang, Xue Wang

**Affiliations:** 1 Department of Dermatology, The First Affiliated Hospital of Shihezi University, Shihezi, China; 2 Shihezi University School Of Medicine, Shihezi, China

**Keywords:** cutaneous metastasis, immunohistochemistry, lung adenocarcinoma, misdiagnosis, Paget ‘ s disease of the breast

## Abstract

**Background:**

Cutaneous metastasis from lung adenocarcinoma is uncommon and may clinically mimic primary dermatologic or breast diseases, particularly when it involves the nipple-areola complex. Such presentations are diagnostically challenging, especially in patients without respiratory symptoms or a smoking history.

**Case presentation:**

We report the case of a 69-year-old woman with no smoking history or pulmonary symptoms who presented with a rapidly enlarging erythematous, edematous, and vesicular plaque involving the left nipple-areola complex and adjacent breast skin. Breast ultrasonography suggested Paget’s disease of the breast, and an initial diagnosis of mammary Paget’s disease was entertained. However, the rapid progression, extensive vesiculation, and absence of an underlying breast mass prompted a skin biopsy. Histopathology revealed dermal infiltration by atypical adenocarcinoma cells. Immunohistochemistry showed positivity for cytokeratin 7, thyroid transcription factor-1, and Napsin A, and negativity for p63 and INSM1, supporting a diagnosis of metastatic lung adenocarcinoma. Chest computed tomography revealed a right upper lobe lung mass with multiple pulmonary metastases, pleural effusion, lymphadenopathy, and suspected bone metastasis. The final diagnosis was stage IVB lung adenocarcinoma with cutaneous metastasis. Molecular testing was declined, and the patient subsequently developed respiratory failure and died four days after intensive care unit admission.

**Conclusion:**

This case underscores that rapidly progressive nipple-areolar eczematous or vesicular lesions should not be presumed to represent Paget’s disease, even in the absence of pulmonary symptoms. Early skin biopsy and multi-marker immunohistochemistry are essential to distinguish cutaneous metastasis from primary breast disease and facilitate timely oncologic evaluation.

## Introduction

The skin represents an uncommon site of distant metastasis in lung cancer, with reported incidence rates ranging from approximately 0.22% to 12%. In lung adenocarcinoma specifically, cutaneous metastasis occurs in approximately 1.58% of cases, most frequently presenting as painless subcutaneous nodules on the chest wall or abdomen (1, 2). When such metastases involve the nipple–areola complex in the absence of respiratory symptoms, they may manifest as erythema, erosion, and crusting—clinically mimicking mammary Paget’s disease—and are therefore susceptible to diagnostic misinterpretation (3). We report the case of a 69-year-old woman without a smoking history or pulmonary symptoms, who initially presented with cutaneous lesions clinically resembling Paget’s disease and was ultimately diagnosed, following skin biopsy and multi-marker immunohistochemical analysis, as having stage IVB right upper lobe lung adenocarcinoma with cutaneous metastases.

## Case report

**Figure 1:**
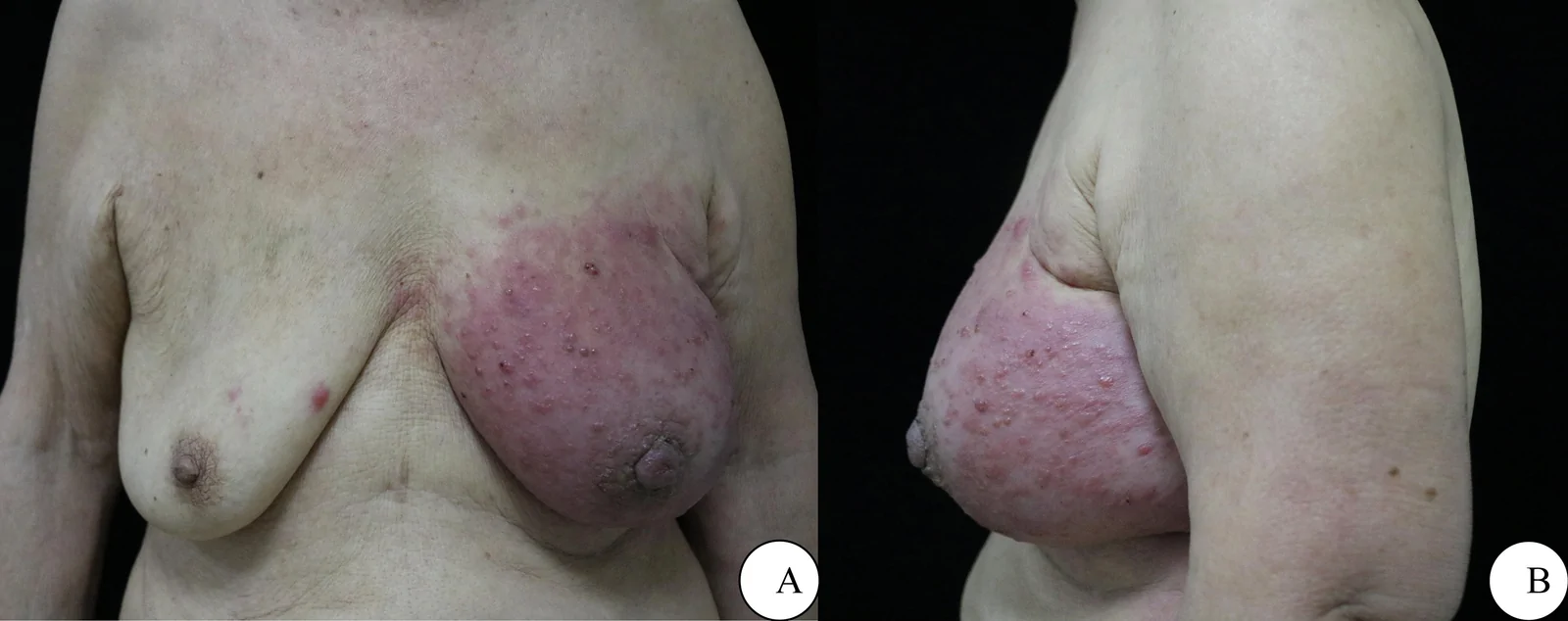
Clinical macroscopic manifestation of cutaneous metastases on a patient’s chest. **(A)** Anterior view exhibiting widespread erythema across the breast and chest wall, accompanied by many papules, nodules, and vesicular lesions; **(B)** Lateral view depicting papules and nodules of diverse sizes merging into patches with a pale crimson surface.

A 69-year-old woman presented to the Dermatology Department in early March 2026 with a 2 cm × 2 cm erythematous plaque involving the left nipple and areola, without identifiable precipitating factors or associated symptoms—including pruritus, pain, or systemic manifestations. By March 15, the lesion had rapidly expanded to 20 cm × 15 cm, presenting as diffuse, edematous erythema with numerous small, translucent vesicles on the surface (**Figure 1**). Throughout the clinical course, the patient consistently denied pruritus, pain, cough, hemoptysis, chest discomfort, or other respiratory symptoms. She had a medical history of hypertension and type 2 diabetes mellitus but denied tobacco use or a family history of malignancy.

On physical examination, a 20 cm × 15 cm well-demarcated, warm, edematous erythematous plaque was noted on the left breast. The surface exhibited scattered papules, papulovesicular lesions, and 1–2 mm vesicles—some of which were ruptured, leading to erosion and crusting. The areolar skin demonstrated thickening, surface irregularity, and infiltrative induration, with overlying dark brown crusts; the nipple architecture remained anatomically preserved. Bilateral axillary lymphadenopathy was present, characterized by palpable, firm, non-mobile lymph nodes.

Laboratory investigations revealed mild anemia and a hypercoagulable profile, with elevated serum levels of IgG and IgA, as well as increased carcinoembryonic antigen (CEA), cytokeratin 19 fragment (CYFRA 21-1), and neuron-specific enolase (NSE). Breast ultrasonography demonstrated skin thickening in the left areolar region with an underlying hyperechoic area, consistent with Paget’s disease of the breast (BI-RADS category 4a), in addition to subcutaneous edema and bilateral axillary lymphadenopathy. A provisional diagnosis of mammary Paget’s disease was established on clinical and imaging grounds.

**Figure 2:**
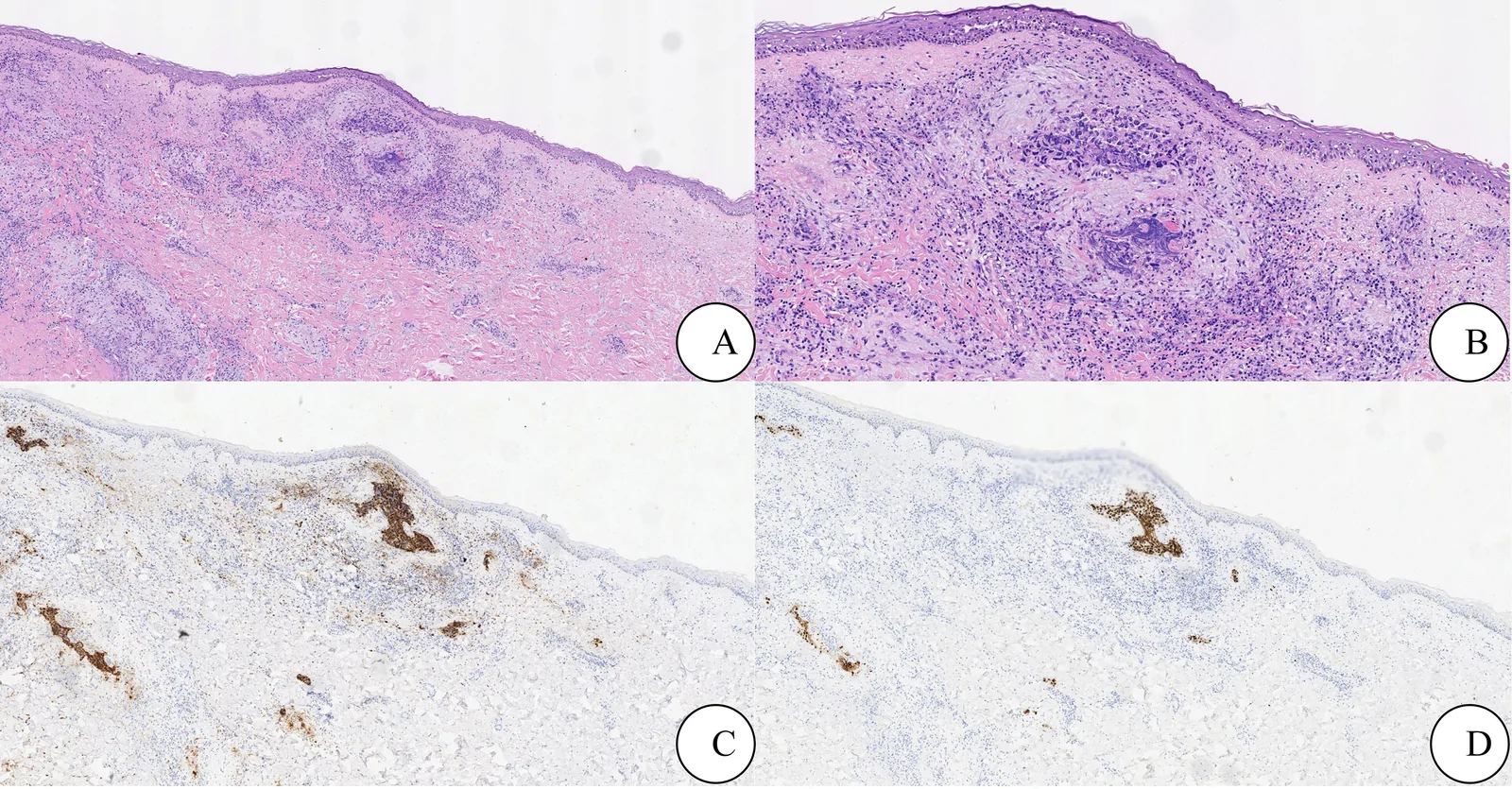
Histopathological and immunohistochemical characteristics of cutaneous metastatic cancer. **(A)** Hematoxylin and eosin (HE) staining (×40); tumor cells infiltrate the dermis in a nests-and-strands configuration, accompanied by inflammatory cell infiltration. **(B)** HE staining (×100); at high magnification, tumor cells exhibit significant atypia, characterized by big, intensely stained nuclei and minimal cytoplasm; glandular-like features are discernible in certain regions. **(C)** Immunohistochemical staining (Napsin A, ×40); tumor cells exhibit positive cytoplasmic expression (brownish-yellow staining); **(D)** Immunohistochemical staining (TTF-1, ×40); tumor cells demonstrate positive nuclear expression (brownish-yellow staining).

Given the atypical clinical features—namely, the widespread edematous erythema with vesiculation, which deviates from the circumscribed eczematous plaques typical of Paget’s disease—and the absence of an identifiable intraductal mass on ultrasonography, a skin biopsy of the left breast was performed. Histopathological examination revealed dermal infiltration by malignant cells arranged in nests and cord-like patterns, accompanied by a lymphohistiocytic inflammatory infiltrate. The tumor cells exhibited marked nuclear atypia, including enlarged, hyperchromatic nuclei and scant cytoplasm; focal glandular differentiation was observed in select areas (**Figures 2A, B**). Immunohistochemical analysis demonstrated positivity for Napsin A, cytokeratin 7 (CK7), and thyroid transcription factor-1 (TTF-1), with negativity for p63 and INSM1; Ki-67 labeling index was approximately 30% (**Figures 2C, D**). This immunophenotypic profile is highly consistent with metastatic lung adenocarcinoma. Consequently, the diagnosis was revised to cutaneous metastasis from pulmonary adenocarcinoma.

A chest computed tomography (CT) scan was obtained to assess for the primary tumor and metastatic burden. It revealed a 72 mm × 56 mm solid mass in the right upper lobe, multiple pulmonary nodules consistent with metastases, left pleural effusion, enlarged mediastinal and bilateral axillary lymph nodes, soft-tissue edema of the left anterior chest wall, and suspected osteolytic lesion in the T11 vertebra. Integrating the clinical, radiological, histopathological, and immunohistochemical evidence, the final diagnosis was stage IVB right upper lobe lung adenocarcinoma with cutaneous metastasis. Molecular profiling was recommended to evaluate eligibility for targeted therapy; however, the patient and her family declined further genetic analysis, precluding initiation of systemic antineoplastic therapy.

Seven days after discharge from the Dermatology service, the patient presented to the Emergency Department with progressive chest tightness and acute-onset wheezing with dyspnea. Owing to rapidly deteriorating respiratory function, she was promptly transferred to the Intensive Care Unit (ICU) and required endotracheal intubation and mechanical ventilation. Empirical antimicrobial therapy with ceftriaxone was initiated, alongside analgesia-sedation (fentanyl and midazolam). Blood cultures subsequently grew methicillin-resistant Staphylococcus aureus (MRSA). Vancomycin was considered for MRSA coverage; however, the family declined further venous access, precluding therapeutic drug monitoring. In view of the patient’s critical condition and the inability to ensure safe vancomycin dosing, the antimicrobial regimen was transitioned to moxifloxacin per the ICU team’s clinical judgment. Despite aggressive supportive measures, respiratory failure progressed irreversibly. The patient expired on April 25, 2026, four days after ICU admission.

## Discussion

Cutaneous metastases from lung cancer account for approximately 7.9%–10% of all metastatic cutaneous neoplasms and typically present as nonspecific nodules or plaques, which may result in misdiagnosis as benign inflammatory conditions or primary breast diseases (4, 5). In this case, the lesion involved the nipple-areola complex and exhibited erythema, erosion, crusting, and eczematous changes, closely resembling mammary Paget’s disease. However, its rapid enlargement, marked edema, and vesiculation over a short period were atypical of the generally indolent course of Paget’s disease (6). Although breast ultrasonography yielded a BI-RADS 4a classification and demonstrated areolar skin thickening, no underlying breast mass was identified, despite the fact that most cases of mammary Paget’s disease are associated with an underlying carcinoma (7). The absence of respiratory symptoms and a smoking history further obscured the diagnosis.

Histopathologically, metastatic lung adenocarcinoma involving the breast skin may exhibit Paget-like epidermal spread or dermal infiltration, making it difficult to distinguish from primary mammary Paget’s disease on the basis of morphology alone (8). Therefore, immunohistochemistry is essential for determining the tumor origin. In the present case, positivity for TTF-1 and Napsin A supported a pulmonary adenocarcinoma origin, whereas negativity for p63 and INSM1 helped exclude squamous and neuroendocrine differentiation (6, 9).

This case emphasizes that rapidly progressive or atypical nipple-areolar lesions should prompt early biopsy, even in patients without respiratory symptoms or a smoking history. Multi-marker immunohistochemistry is critical for differentiating cutaneous metastasis from primary breast disease. Because cutaneous metastasis from lung cancer generally indicates advanced disease and a poor prognosis, with a reported median survival of approximately 8 months, timely recognition is important for guiding staging, molecular testing, and treatment planning (5, 10). In the present case, refusal of genetic testing and rapid respiratory deterioration limited systemic treatment, further underscoring the need for early diagnosis and multidisciplinary evaluation.

## Data Availability

The original contributions presented in the study are included in the article/**Supplementary Material**. Further inquiries can be directed to the corresponding authors.
